# Clinical significance of TLR7/IL-23/IL-17 signaling pathway in patients with acute respiratory distress syndrome

**DOI:** 10.1016/j.clinsp.2024.100358

**Published:** 2024-09-11

**Authors:** Lihong Chu, Fengqi Liu, Kankai Tang

**Affiliations:** The First People's Hospital of Huzhou, Huzhou, Zhejiang, PR China

**Keywords:** Acute respiratory distress syndrome, Toll-like receptor 7, Interleukin-23, Interleukin-17

## Abstract

**Objectives:**

To analyze the clinical significance of Toll-Like Receptor 7/Interleukin-23/Interleukin-17 (TLR7/IL-23/IL-17) signaling pathway in patients with Acute Respiratory Distress Syndrome (ARDS).

**Method:**

The clinical data of 85 patients with ARDS were retrospectively analyzed and set as the ARDS group, and the clinical data of 85 healthy participants during the same period were set as the healthy control group. Univariate and multivariate logistic regression were used to analyze risk the factors affecting the prognosis of ARDS patients.

**Results:**

TheTLR7 mRNA expression and IL-23 and IL-17 levels in peripheral blood mononuclear cells were higher in the ARDS group than in the control group (p < 0.05). TLR7 mRNA expression, IL-23, IL-17, Surfactant Protein-D (SP-D), and Clara Cell protein-16 (CC-16) levels were the highest in the severe group, followed by the moderate group, and the lowest in the mild group, while Oxygenation Index (OI) was the lowest in the severe group, followed by the moderate group, and the highest in the mild group (p < 0.05). Multivariate logistic regression analysis found that the disease grade (severe), TLR7 mRNA expression, IL-23 level, and IL-17 level were the risk factors affecting the 28-d survival status of ARDS patients (OR > 1, p < 0.05).

**Conclusions:**

In ARDS patients, the TLR7/IL-23/IL-17 signaling pathway is activated. The expression of this pathway is closely related to the severity of the disease and the levels of lung injury markers, and it is a risk factor that may have a direct impact on the prognosis of ARDS patients.

## Highlights

• TLR7/IL-23/IL-17 pathway activation in ARDS patients implicates immune dysregulation in disease severity.
• TLR7/IL-23/IL-17 pathway is associated with adverse short-term prognosis in ARDS.
• SP-D and CC-16 are positively correlated with TLR7/IL-23/IL-17 pathway activation, indicating lung injury involvement in ARDS progression.

## Introduction

Acute progressive respiratory failure, also known as Acute Respiratory Distress Syndrome (ARDS), is caused by non-cardiogenic pulmonary and intrapulmonary factors, including trauma, shock, severe infection, and burns.^1^ The disease has immediate onset and complex etiology, with predominant features being respiratory distress and uncontrollable hypoxemia. Hypoxic symptoms such as cyanosis and accelerated respiration are also common.^2^,^3^ Epidemiological studies indicate that China has an annual ARDS incidence of 59/100,000; with 670,000 new cases recorded each year and a fatality rate of 40%–50%.^4^ Currently, patient imaging, oxygenation, and clinical features are mostly combined for clinical diagnosis of ARDS and illness assessment. However, some tests exhibit lag that generates challenges for accurate prognostic predictions. The development of serum biochemical indices with high sensitivity and specificity is thus urgently necessary to aid in the clinical diagnosis and treatment of ARDS.

The pathogenesis of multi-organ dysfunction syndrome is linked to an uncontrolled inflammatory response.^5^,^6^ In particular, ARDS pathophysiology involves an unregulated inflammatory response that impairs the functions of the alveolar epithelium and pulmonary vascular endothelium.^7^ Alveolar epithelial cells can be divided into type I and type II, both of which are damaged during diffuse alveolar injury. Common lung injury markers include Surfactant Protein-D (SP-D) and Clara Cell protein-16 (CC-16). Although the specifics remain unknown, ARDS pathogenesis is generally believed to be associated with immune dysregulation and imbalance.^8^ During acute lung injury, exogenous factors stimulate alveolar macrophages and cause inflammatory cytokine synthesis, in turn triggering the production of numerous proteases, chemokines, and oxygen-free radicals. These compounds contribute to the formation of pulmonary hyaline membranes and increase pulmonary capillary permeability, ultimately inducing ARDS. Simultaneously, an acute uncontrolled inflammatory response can produce pulmonary edema; this condition causes edematous fluid rich in inflammatory cells and proteins to enter the pulmonary interstitial and alveolar spaces, increasing ARDS risk.

Toll-Like Receptors (TLRs) identify pathogenic microbes and may function as the first line of defense in lung inflammatory response. They also regulate the expression of downstream inflammatory factors. In particular, Toll-Like Receptor 7 (TLR7) is broadly distributed on immune cell membranes, contributing to lung damage via identifying features specific to bacteria and viruses, thus activating airway responses.^9^,^10^ During pathogen invasion, TLR7 triggers plasmacytoid dendritic cells to secrete Interleukin (IL) factors (e.g., IL-23 and IL-17) through MyD88-independent and MyD88-dependent signaling pathways. These processes drive Th1 cell immunity, activating antigen-specific cytotoxic T-cells, phagocytes, and other cells to release cytokines. Notably, an imbalance in the ratio of pro-inflammatory helper T-cell 17 and anti-inflammatory regulatory T-cells (Th17/Treg) increases the risk of poor prognosis in ARDS. This imbalance is reflected in the concentrations of cytokines IL-23 and IL-17.^11^ IL-23 can specifically bind to the surface receptors of effector T-cells such as CD4+, inducing IL-17 production. This contribution to the inflammatory response maintains normal Th17 cell differentiation and function. Here, the authors hypothesized that, along with driving Th1 cells to participate in immune and inflammatory responses, TLR7 also exacerbates those responses by activating the IL-23/IL-17 signaling pathway. To analyze the role of the TLR7/IL-23/IL-17 signaling pathway in ARDS occurrence, development, and prognosis, the authors first compared the expression of the TLR7/IL-23/IL-17 signaling pathway between patients and healthy individuals. Next, the authors analyzed how pathway activation is related to the symptoms, SP-D, CC-16, and prognosis of ARDS patients.

## Materials and methods

### Clinical data

This study retrospectively selected 85 patients with ARDS who were admitted to the hospital from March 2019 to June 2022. The sample included 50 males and 35 females, ranging in age from 32 to 79 years (mean = 57.9 ± 10.3-years), with varying degrees of disease severity (mild = 29 cases, moderate = 41, severe = 15). Additionally, a healthy control group was formed of 85 individuals who participated in check-ups at the hospital during the same period. This group included 47 males and 38 females aged 35–79 years (mean = 58.4 ± 9.5 years).

### Criteria for the ARDS group

#### Diagnostic criteria and disease severity

The diagnostic criteria for ARDS were as follows: meeting the Berlin definition proposed by the European Society of Intensive Care Medicine in 2012^12^; onset time of new or worsening respiratory symptoms within 1-week; chest Computed Tomography (CT) or chest radiograph showing patchy and blurred shadow of both lungs that cannot be explained by pulmonary nodules, pulmonary atelectasis, or pleural effusion; respiratory failure from pulmonary edema not fully explained by volume overload or heart failure. Disease severity was split into mild (Oxygenation Index [OI] > 200 mmHg and ≤ 300 mmHg), moderate (OI > 100 mmHg and ≤ 200 mmHg), and severe (OI ≤ 100 mmHg).

#### Inclusion and exclusion criteria

Patients with the following conditions were included: meeting ARDS diagnostic criteria at any level of severity; were between 18 to 80 years old (inclusive); exhibited clear etiology, including acute pancreatitis, trauma, and severe pneumonia; experienced tracheal intubation or tracheotomy connected to ventilator-assisted ventilation; and complete clinical data were available. Patients were excluded if they met the following conditions: received immunosuppressant or hormone therapy 6-months before enrollment; were diagnosed with autoimmune diseases, neoplastic diseases, blood system diseases, chronic liver diseases, congenital heart disease or other acute and chronic inflammatory diseases, as well as respiratory diseases or other lung diseases; died within 24 hours after admission; lost to follow-up; pregnant or breast-feeding. This study was approved by the Ethics Committee of The First People's Hospital of Huzhou (n° 2022KYLL041) and complied with the Declaration of Helsinki. The study was a retrospective analysis and followed the STARD guidelines.

### Sample collection

On the day of the physical examination of healthy controls and before the ARDS group received treatment, 4 mL of peripheral venous blood was collected from all participants and divided into two equal portions. One portion was placed in an anticoagulant tube containing EDTA to isolate mononuclear cells from peripheral blood using Ficoll density gradient centrifugation. The other portion was centrifuged (2000 r/min, *R* = 6 cm) for 20 min to obtain the supernatant, which was stored at -80°C until use.

### TLR7 mRNA in peripheral blood mononuclear cells

Total RNA was extracted from peripheral blood mononuclear cells using a Trizol kit and Total RNA Extraction Kit (kit purchased from Thermo Fisher Scientific). The RNA concentration was determined using a spectrophotometer (NanoDrop ND-1000, NanoDrop, USA), reverse-transcribed into cDNA (Yisheng Biotechnology Co., Ltd., Shanghai), and stored at -20°C. TLR7 expression was measured using qRT-PCR (Shanghai Hongshi Medical Technology Co., Ltd.), with cDNA as the template. The reaction volume was 20 μL, and the thermocycling schedule was as follows: 95°C for 2 min; 40 cycles of 95°C for 5s and 60°C for 30s; 72°C for 3-min. Fluorescence data was collected at 72°C for each cycle, and TLR7 expression was calculated with the 2^−ΔΔCt^ method. The internal reference was U6. The following primers were used: forward 5ʹ-GTCGTTCGGCAGCACA-3ʹ, reverse 5ʹ-AACGCTTCACGAATTTGCGT-3ʹ (U6); forward 5ʹ-CCCCATTCCTTGTGCGCCG-3ʹ, reverse 5ʹ- ACCATCTTGGGGCACATGCT3-3ʹ (TLR7).

### Levels of serum IL-23, IL-17, and lung injury markers

Levels of serum IL-23, IL-17, SP-D, and CC-16 were determined from thawed serum samples using Enzyme-Linked Immunosorbent Assay (ELISA) kits (Shanghai Lepisit Biotechnology).

### Short-term prognosis

Patients were followed up for 28d after admission to the hospital. Deaths within 28d were counted to divide participants into death and survival groups.

### Statistical analysis

All data were analyzed in SPSS 23.0 software. Measurement data conforming to the normal distribution were represented as means ± standard deviation and subjected to *t*-tests. Count data were represented as percentages and subjected to χ^2^ tests. Relationships between TLR7, IL-23, and IL-17 levels with OI and lung injury markers were determined using Spearman rank correlations. Receiver Operator Characteristic (ROC) curves were drawn to analyze the predictive value of TLR7, IL-23, and IL-17, separately and in combination. Multivariate logistic regression was used to analyze the risk factors affecting patient prognosis. Significance was set at p < 0.05.

## Results

### Demographic data of ARDS and control groups

Sex, age, and body mass index did not differ between the ARDS and control groups (p > 0.05) (Table 1).

**Table 1:** Comparison of demographic data of ARDS and control groups (n, mean ± SD).

					Pathogenic factors	Underlying disease	Adverse habits
Group	Number of cases	Sex (Male/ Female)	Age (years)	Body mass index (kg/m^2^)	Acute pancreatitis/ Trauma/ Severe pneumonia/ Other	Hypertension/ Diabetes/ Hyperlipidemia/ Coronary heart disease	History of smoking/ Alcohol consumption
Control group	85	47/38	58.4±9.5	24.84±2.29	‒	‒	‒
ARDS group	85	50/35	57.9±10.3	24.37±2.08	10/25/48/2	22/15/23/11	27/19

ARDS, Acute Respiratory Distress Syndrome.

### Comparisons of TLR7, IL-23, IL-17 levels between ARDS and control groups

Peripheral blood TLR7 mRNA expression, IL-23, and IL-17 levels in peripheral blood mononuclear cells were higher in the ARDS group than in the control group (p < 0.05) (Table 2).

**Table 2:** Comparison of TLR7, IL-23 and IL-17 levels in peripheral blood mononuclear cells between ARDS and control groups (mean ± SD).

Group	Number of cases	TLR7 mRNA	IL-23 (pg/mL)	IL-17 (pg/mL)
Control group	85	0.96 ± 0.13	95.95 ± 17.44	17.10 ± 3.29
ARDS group	85	1.30 ± 0.20^a^	123.01 ± 19.59^a^	22.90 ± 4.04^a^

Note: Compared with the control group

a p < 0.001. TLR7, Toll-Like Receptor 7; IL-23, Interleukin-23; IL-17, Interleukin-17; ARDS, Acute Respiratory Distress Syndrome.

### Predictive value of TLR7, IL-23, IL-17 levels for ARDS

**Fig. 1:**
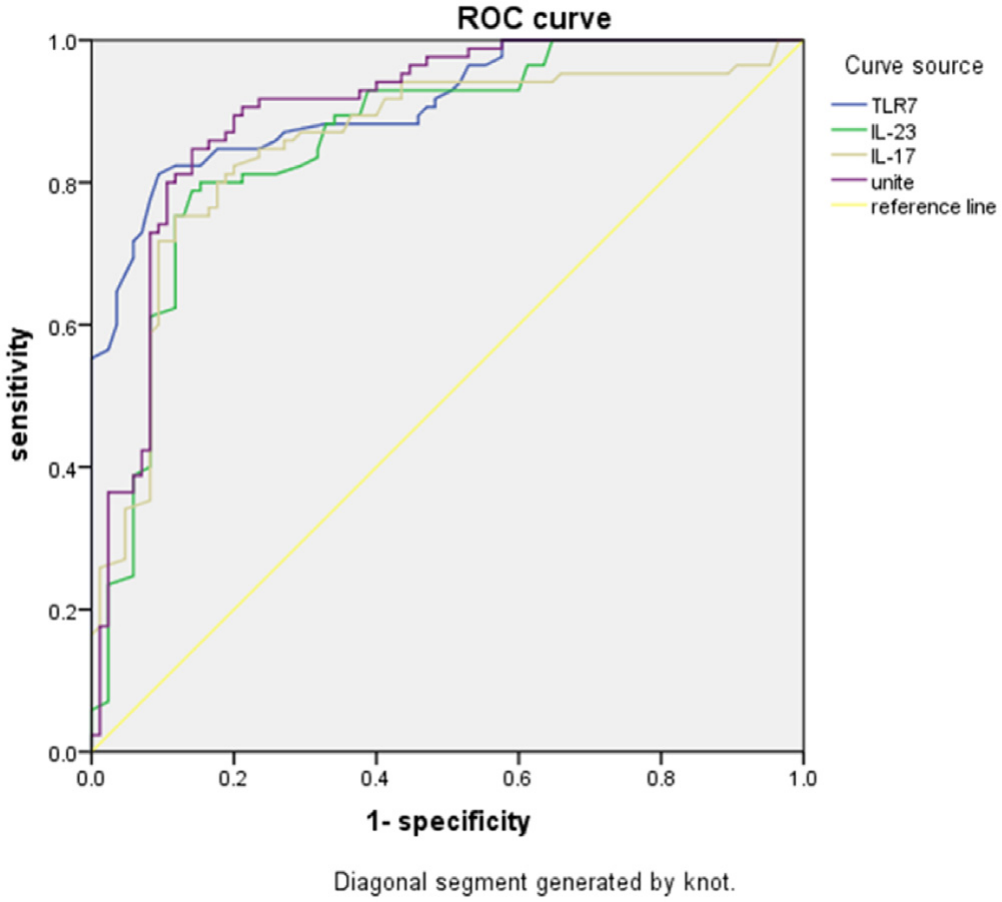
ROC plots of TLR7, IL-23, and IL-17 levels and their combined predictive value for ARDS. TLR7, Toll-Like Receptor 7; IL-23, Interleukin-23; IL-17, Interleukin-17; ARDS, Acute Respiratory Distress Syndrome.

Whether ARDS occurred or not was used as a status variable (0 = not occurred, 1 = occurred), and TLR7 mRNA expression and IL-23 and IL-17 levels in peripheral blood mononuclear cells were used as test variables to plot the ROC curve. The results of the ROC analysis showed that with 1.158 as the best cut-off value, the Youden index of TLR7 mRNA expression for predicting ARDS was 0.537, and the Area Under the Curve (AUC) was 0.899. With 103.655 pg/mL as the best cut-off value, the Youden index of IL-23 for predicting ARDS was 0.475, and the AUC was 0.860. With 20.47 pg/mL as the best cut-off value, the Youden index of IL-17 for predicting ARDS was 0.444, and the AUC was 0.854. The Youden index of the combination of expression of TLR7 mRNA in peripheral blood mononuclear cells and the levels of IL-23 and IL-17 for predicting ARDS was 0.612, and the AUC was 0.911 (Table 3 and Fig. 1).

**Table 3:** Predictive value of TLR7, IL-23, IL-17 levels for ARDS.

Indicator	Best cut-off value	Standard error	Sensitivity	Specificity	Youden Index	AUC	95% CI
TLR7 mRNA	1.158	0.025	82.02%	71.69%	0.537	0.899	0.850‒0.947
IL-23	103.655pg/mL	0.029	78.56%	68.95%	0.475	0.860	0.803‒0.917
IL-17	20.47pg/mL	0.031	77.15%	67.28%	0.444	0.854	0.793‒0.914
Combination	‒	0.022	85.36%	75.85%	0.612	0.911	0.868‒0.953

TLR7, Toll-Like Receptor 7; IL-23, Interleukin-23; IL-17, Interleukin-17; ARDS, Acute Respiratory Distress Syndrome; AUC, Area Under the Curve.

### TLR7, IL-23, IL-17 levels in patients with differing ARDS severity

**Fig. 2:**
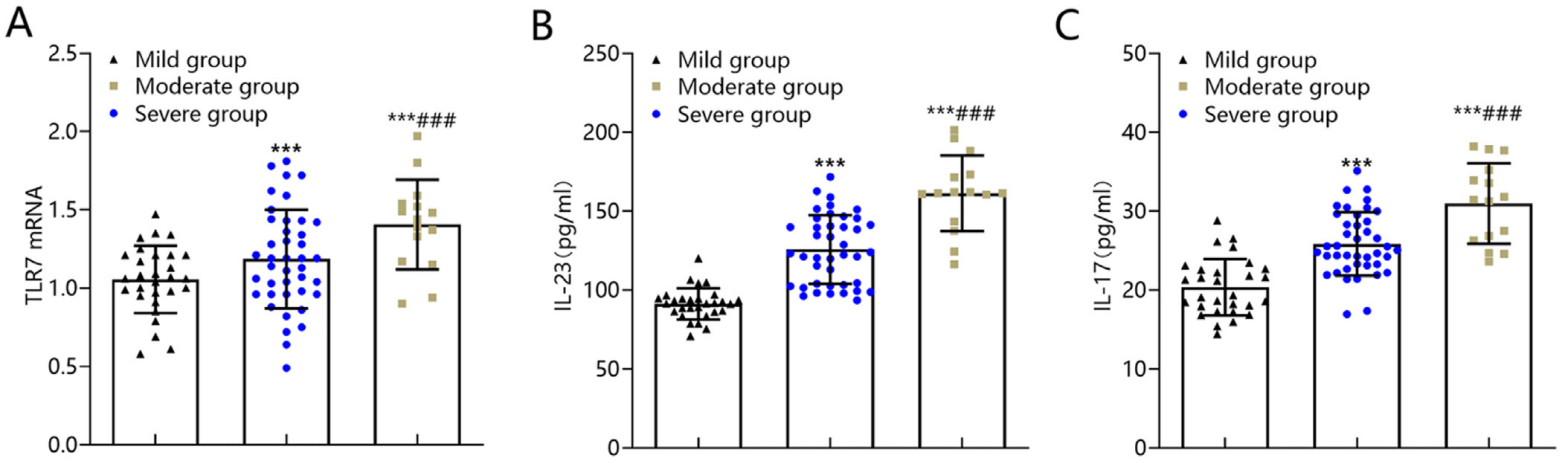
Comparison of TLR7, IL-23 and IL-17 levels in peripheral blood mononuclear cells of patients with different conditions in ARDS group. The expression of TLR7 mRNA (A) in peripheral blood mononuclear cells and the levels of IL-23 (B) and IL-17 (C) in the severe group were significantly higher than those in moderate and mild groups. Note: Compared with the mild group, ^⁎⁎⁎^p < 0.001; compared with the moderate group, ^###^p < 0.001. TLR7, Toll-Like Receptor 7; IL-23, Interleukin-23; IL-17, Interleukin-17; ARDS, Acute Respiratory Distress Syndrome.

The expression of TLR7 mRNA in peripheral blood mononuclear cells and the levels of IL-23 and IL-17 were the highest in the severe group, followed by the moderate group, and the lowest in the mild group (p < 0.05) (Fig. 2).

### OI and lung injury markers in patients with differing ARDS severity

**Fig. 3:**
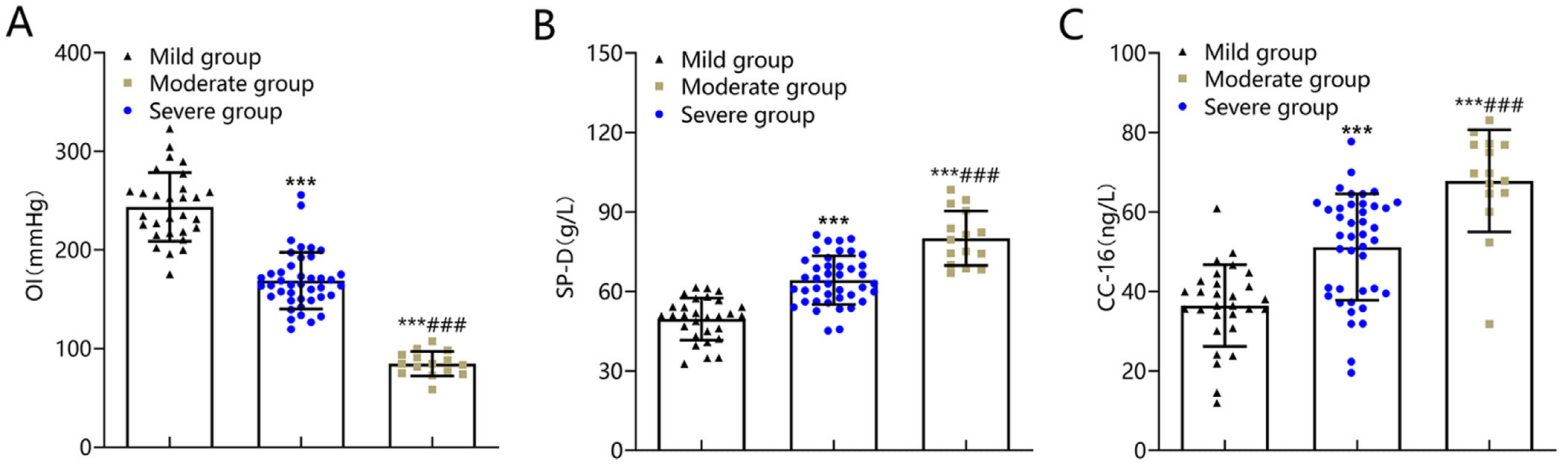
Comparison of OI and lung injury marker levels in patients with different conditions in the ARDS group. The OI (A) of the severe group was significantly lower than that of the moderate and mild groups, and the serum SP-D (B) and CC-16 (C) levels were significantly higher than those of the moderate and mild groups. Note: Compared with mild group, ^⁎⁎⁎^p < 0.001; compared with moderate group, ^###^p < 0.001. ARDS, Acute Respiratory Distress Syndrome; OI, Oxygenation Index; SP-D, Surfactant Protein-D; CC-16, Clara Cell protein-16.

OI was the lowest in the severe group, followed by the moderate group, and the highest in the mild group, while serum SP-D and CC-16 levels were the highest in the severe group, followed by the moderate group, and the lowest in the mild group (p < 0.05) (Fig. 3).

### Correlation analysis of TLR7, IL-23, IL-17 levels with OI and lung injury markers

TLR7 mRNA, IL-23, and IL-17 levels in peripheral blood mononuclear cells were negatively correlated with OI (Spearman's *r* < 0, p < 0.05) and positively correlated with SP-D and CC-16 levels (Table 4).

**Table 4:** Correlation analysis of TLR7, IL-23, IL-17 levels with OI and lung injury markers.

Indicator	Coefficient	OI	SP-D	CC-16
TLR7 mRNA	*r*	−0.636	0.586	0.702
	p	0.000	0.000	0.000
IL-23	*r*	−0.619	0.572	0.657
	p	0.000	0.000	0.000
IL-17	*r*	−0.592	0.538	0.635
	p	0.000	0.000	0.000

TLR7, Toll-Like Receptor 7; IL-23, Interleukin-23; IL-17, Interleukin-17; OI, Oxygenation Index; SP-D, Surfactant Protein-D; CC-16, Clara Cell protein-16.

### Baseline data of ARDS patients with different prognoses

**Fig. 4:**
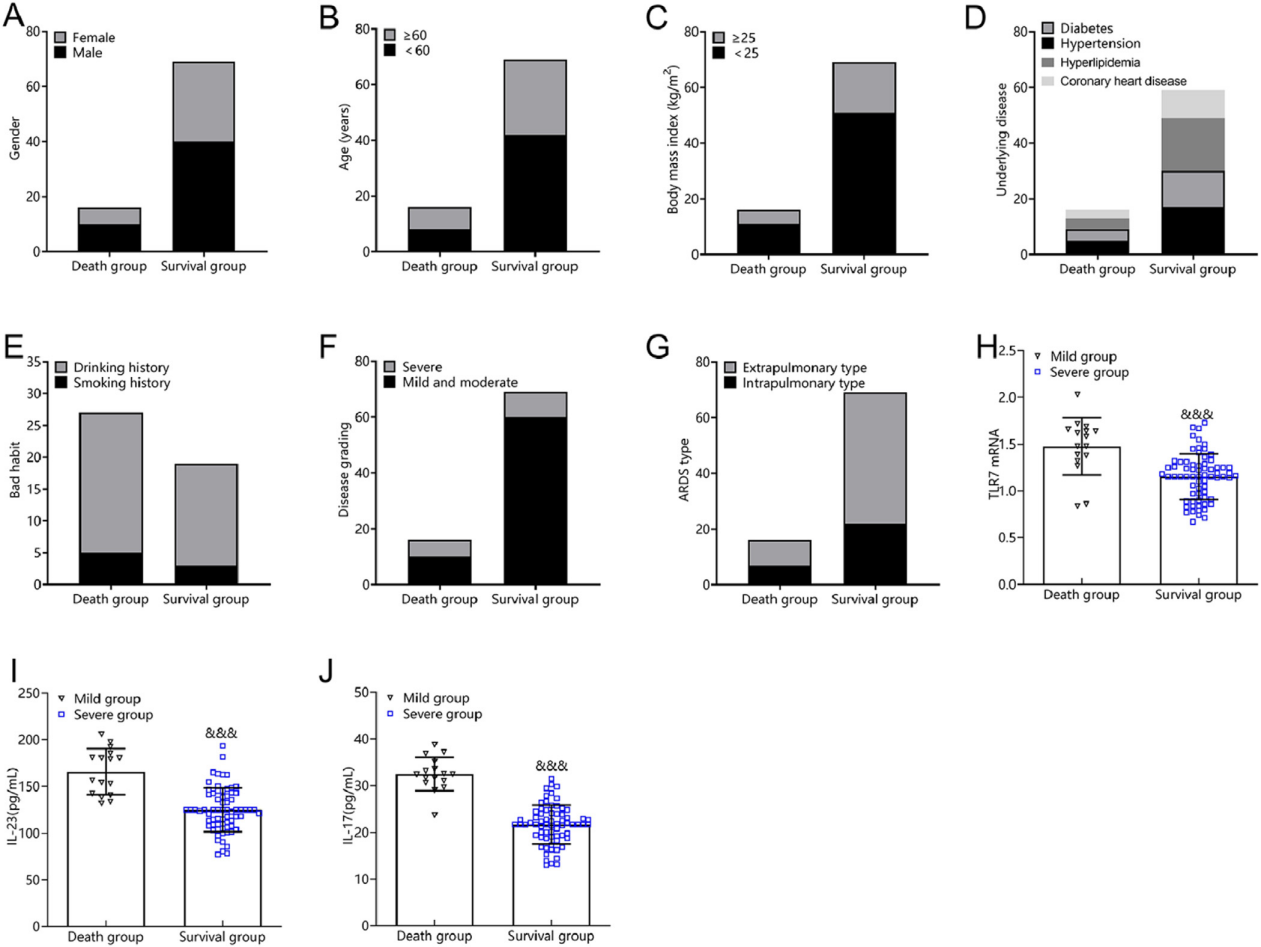
Comparison of baseline data of patients with different prognosis in ARDS group. (A) Gender; (B) Age; (C) Body mass index; (D) Underlying disease; (E) Adverse habits; (F) Disease classification; (G) Type of ARDS; (H) TLR7 mRNA; (I) IL-23; (J) IL-17. Note: Compared with the death group, ^&&&^ p < 0.001.

Sex, age, body mass index, underlying disease, bad habits, and percentage of ARDS types did not differ between death and survival groups (p > 0.05). Patients who died exhibited a higher percentage of severe ARDS than patients who survived, while also having higher TLR7 mRNA, IL-23, and IL-17 levels (p < 0.05) (Fig. 4).

### Logistic multivariate analysis of factors affecting the prognosis

The 28-d survival status of ARDS patients was used as the dependent variable ("1" = death, "0" = survival), and the variables with statistically significant differences were used as the independent variables. Multivariate logistic regression revealed that disease stage (severe), TLR7 mRNA expression, IL-23 levels, and IL-17 levels were risk factors affecting the 28-d survival status of ARDS patients (OR > 1, p < 0.05) (Table 5).

**Table 5:** Logistic multivariate analysis of risk factors affecting the prognosis of ARDS patients.

Factor	β	SE	Wald *χ^2^*	p	OR	95% CI
Disease classification (severe)	1.386	0.628	4.872	0.027	4.000	1.168‒13.698
TLR7 mRNA	1.216	0.535	5.262	0.023	3.402	1.189‒9.862
IL-23	1.168	0.524	4.964	0.026	3.215	1.142‒8.986
IL-17	1.179	0.569	4.312	0.035	3.202	1.068‒9.897
Constant	-5.021	1.035	27.462	0.000	0.004	‒

TLR7, Toll-Like Receptor 7; IL-23, Interleukin-23; IL-17, Interleukin-17; ARDS, Acute Respiratory Distress Syndrome.

## Discussion

Th17/Treg cells are a pair of pro-inflammatory and anti-inflammatory immune regulatory cells. The former secretes large amounts of IL-17 after activation, directly increasing alveolar epithelial permeability. Animal experimental results ^13^ demonstrated that the Treg/Th17 ratio was unbalanced in ARDS rats, while cytokines such as IL-17 and IL-6 increased. These findings indicate that ARDS rats are associated with immune dysregulation. Similarly, Ding et al.^14^ found that serum IL-17 levels increased significantly in ARDS. Specifically, lung injury scores were reduced in mice 48h after administering an IL-17-blocking antibody. The antibody successfully alleviated symptoms of acute respiratory infection via affecting retinoic acid Receptor-related Orphan Receptor gamma-t (RORγt) levels and the PI3K pathway.

The immune molecule TLR7 elevates Th17 cells and is involved in airway inflammation and immune imbalance through secreting IL-17, as well as recruiting and activating neutrophils.^15^,^16^ Additionally, IL-23 binds specifically to effector T-cell surface receptors such as CD4+ to induce IL-17 production, which participates in the inflammatory response and maintains normal Th17 cell differentiation and function. Thus, a commonly accepted hypothesis is that TLR7, while driving Th1 cell immunity, may also activate the IL-23/IL-17 signaling pathway by stimulating Th17 cell immunity and exacerbating the inflammatory response. Previous research in older patients has confirmed that TLR7/IL-23/IL-17 signaling activation is associated with lung infections,^17^ but currently, the authors have no clear evidence regarding whether this pathway is associated with ARDS.

This study found that TLR7 mRNA expression, along with IL-23 and IL-17 levels, were higher in ARDS peripheral blood than in healthy controls. Furthermore, the levels of these indicators were negatively correlated with OI. The ROC curves further revealed that the Youden index of the combination of the three indicators was 0.612, with an AUC of 0.911. These values indicate that the TLR7/IL-23/IL-17 signaling pathway is activated in patients with ARDS, and its expression is correlated with the severity of the disease. The present results suggest that clinical monitoring of changes in TLR7/IL-23/IL-17 signaling should be performed regularly. Moreover, targeted measures should be taken to downregulate pathway expression as a method to alleviate symptoms.

The results of the logistic regression revealed that TLR7 mRNA expression, IL-23 levels, and IL-17 levels were risk factors affecting patient survival at 28d. These results confirm that the activation of the TLR7/IL-23/IL-17 signaling pathway increases the risk of adverse short-term prognosis in ARDS. Several factors may explain these results. First, abnormally elevated levels of TLR7, IL-23, and IL-17 activate the systemic inflammatory response, causing neutrophils to accumulate in the lungs. This accumulation destroys epithelial and endothelial integrity, further expanding the alveolar-arterial oxygen gradient and increasing cell permeability. The outcome is interstitial pulmonary edema, along with negative effects on oxygenation function and prognosis. Second, strong activation of the specific immune response aggravates inflammatory damage in the liver and elevates inflammatory cytokines. These factors subsequently activate the TLR7/IL-23/IL-17 signaling pathway, which is clearly critical to determining ARDS prognosis.

CC16 is secreted by Clara cells distributed in the fine bronchial mucosa of the lungs. Its primary function is to protect the pulmonary surfactant from degradation and remove harmful substances deposited in the respiratory tract. In addition, CC16 exhibits antifibrotic, antioxidant, antitumor, and anti-inflammatory properties. Increased serum CC16 levels reflected impaired integrity of the alveolar epithelium and vascular endothelium.^18^ In patients with severe chest trauma, serum CC16 levels tended to increase during the immediate post-traumatic period and then decreased to a normal range after 12–24h.^19^ As a protein in the collagen agglutination family, SP-D is synthesized and secreted by alveolar type II epithelial cells and airway Clara cells. It mediates the immune response and inflammatory reaction and is involved in maintaining alveolar physiological function and inhibiting oxidative stress.^20^ Studies have demonstrated that abnormally elevated SP-D levels are linked with the pathogenesis of lung injury-related diseases such as severe pneumonia and chronic obstructive pulmonary disease.^21^,^22^ In this study, serum SP-D and CC-16 levels were the highest in the severe group, followed by the moderate group, and the lowest in the mild group, and their levels were positively correlated with TLR7 mRNA, IL-23, and IL-17 levels. The authors thus speculated that TLR7/IL-23/IL-17 pathway activation may be involved in ARDS development and progression, specifically via aggravating lung injury.

## Conclusion

In conclusion, activating the TLR7/IL-23/IL-17 signaling pathway in ARDS patients is closely related to the severity of the disease and the levels of lung injury markers (SP-D and CC-16) and are risk factors affecting the short-term prognosis of ARDS patients. However, this study has several limitations, notably the small sample size, geographical limitations of case sources, and short follow-up time. Future studies should therefore aim to increase the number of participants, include multicenter study subjects, expand the range of case sources, and extend the follow-up period.

### Authors’ contributions

All authors designed experiments, carried out experiments, and analyzed experimental results. Lihong Chu wrote the manuscript. Kankai Tang revised the manuscript. All authors approved the final manuscript.

### Funding

This research did not receive any specific grant from funding agencies in the public, commercial, or not-for-profit sectors.

## Declaration of competing interest

The authors declare no conflicts of interest.
